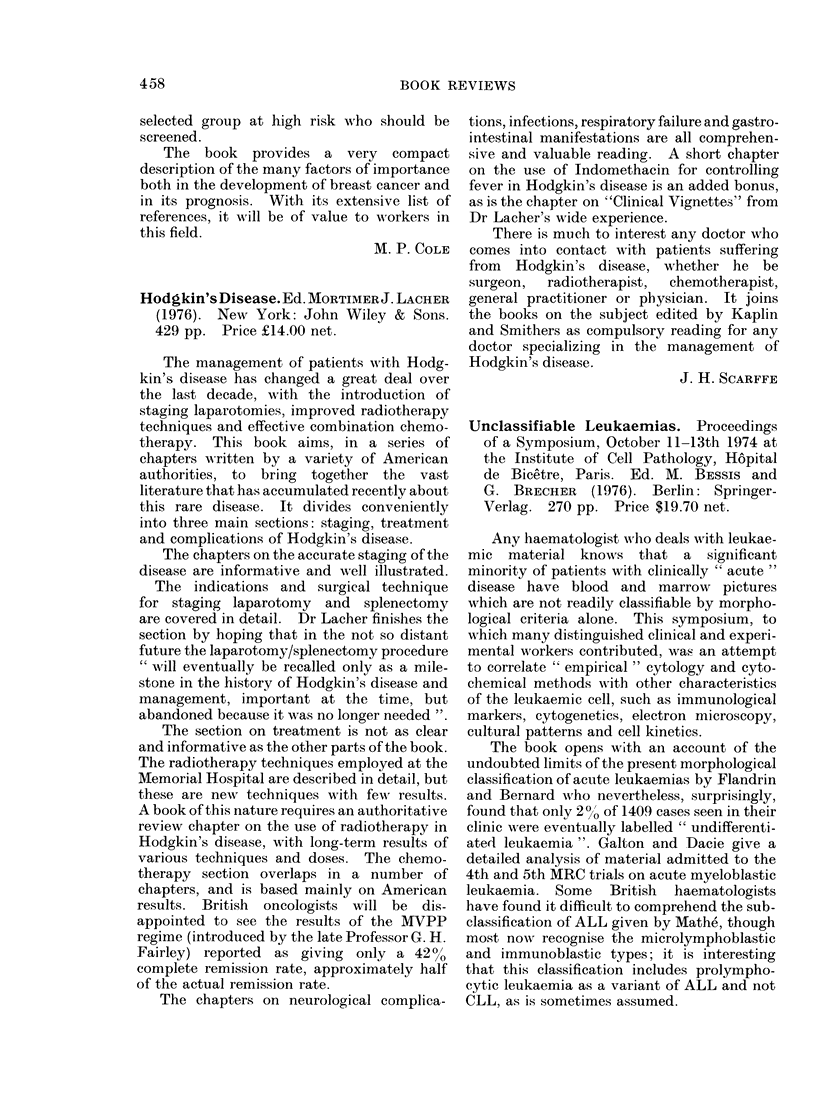# Hodgkin's Disease

**Published:** 1976-10

**Authors:** J. H. Scarffe


**Hodgkin's Disease.** Ed. Mortimer J. Lacher (1976). New York: John Wiley & Sons. 429 pp. Price £14.00 net.

The management of patients with Hodgkin's disease has changed a great deal over the last decade, with the introduction of staging laparotomies, improved radiotherapy techniques and effective combination chemotherapy. This book aims, in a series of chapters written by a variety of American authorities, to bring together the vast literature that has accumulated recently about this rare disease. It divides conveniently into three main sections: staging, treatment and complications of Hodgkin's disease.

The chapters on the accurate staging of the disease are informative and well illustrated. The indications and surgical technique for staging laparotomy and splenectomy are covered in detail. Dr Lacher finishes the section by hoping that in the not so distant future the laparotomy/splenectomy procedure " will eventually be recalled only as a milestone in the history of Hodgkin's disease and management, important at the time, but abandoned because it was no longer needed ".

The section on treatment is not as clear and informative as the other parts of the book. The radiotherapy techniques employed at the Memorial Hospital are described in detail, but these are new techniques with few results. A book of this nature requires an authoritative review chapter on the use of radiotherapy in Hodgkin's disease, with long-term results of various techniques and doses. The chemotherapy section overlaps in a number of chapters, and is based mainly on American results. British oncologists will be disappointed to see the results of the MVPP regime (introduced by the late Professor G. H. Fairley) reported as giving only a 42% complete remission rate, approximately half of the actual remission rate.

The chapters on neurological complications, infections, respiratory failure and gastrointestinal manifestations are all comprehensive and valuable reading. A short chapter on the use of Indomethacin for controlling fever in Hodgkin's disease is an added bonus, as is the chapter on "Clinical Vignettes" from Dr Lacher's wide experience.

There is much to interest any doctor who comes into contact with patients suffering from Hodgkin's disease, whether he be surgeon, radiotherapist, chemotherapist, general practitioner or physician. It joins the books on the subject edited by Kaplin and Smithers as compulsory reading for any doctor specializing in the management of Hodgkin's disease.

J. H. Scarffe